# Dynamic Uncertainty for Compensated Second-Order Systems

**DOI:** 10.3390/s100807621

**Published:** 2010-08-13

**Authors:** Sascha Eichstädt, Alfred Link, Clemens Elster

**Affiliations:** Physikalisch-Technische Bundesanstalt, Abbestr. 2-12, 10587 Berlin, Germany; E-Mails: alfred.link@ptb.de (A.L.); clemens.elster@ptb.de (C.E.)

**Keywords:** sensor, dynamic uncertainty, digital filter, deconvolution

## Abstract

The compensation of LTI systems and the evaluation of the according uncertainty is of growing interest in metrology. Uncertainty evaluation in metrology ought to follow specific guidelines, and recently two corresponding uncertainty evaluation schemes have been proposed for FIR and IIR filtering. We employ these schemes to compare an FIR and an IIR approach for compensating a second-order LTI system which has relevance in metrology. Our results suggest that the FIR approach is superior in the sense that it yields significantly smaller uncertainties when real-time evaluation of uncertainties is desired.

## 1. Introduction

Various important types of sensors like accelerometers or load cells can be modeled by a mass-spring system resulting in a second-order model of the kind:

(1) $$H(s)=\frac{S_{0}\omega_{0}^{2}}{s^{2}+2\delta \omega_{0}s+\omega_{0}^{2}},$$

where *S*_0_, *δ* and *ω*_0_ = 2*π f*_0_ denote static gain, damping and resonance frequency, see [1–4]. When such sensors are applied for the measurement of according signals with significant frequency content near the resonance frequency the sensor output signal contains time-dependent distortions such as ringing. Analogue and digital filtering are appropriate tools to reduce these dynamic errors by compensating the dynamic response of the sensor, and techniques for the construction of compensation filters are well-known in digital signal processing (DSP), see, for instance, [1–3,5–8].

The model parameters in (1) are usually not known from the start, but need to be determined by system identification using calibration measurements, see [4,9] for the example of an accelerometer identification. Due to the uncertainty of the calibration measurements, the identified system is also uncertain to some extent. For a complete assessment of the compensation quality this uncertainty may not always be ignored. The treatment of this uncertainty and the deconvolution of uncertain systems is a broad topic in DSP, mainly in the field of robust filtering and control [10–12].

Metrology is another field with a recently growing interest in the compensation of uncertain dynamic systems [13–21]. As metrology is concerned with the establishment of measurement units, the realization of measurement standards and the transfer of traceability from these standards to industry, measurements at the highest level of accuracy are aimed at. Furthermore, a standardized assessment of the uncertainty associated with the measurement result is important. The uncertainty needs to include all relevant influences, and in the context of dynamic measurements the uncertainty of a designed compensation filter (caused by the uncertain knowledge of the underlying dynamic system) has to be accounted for. The basis for the standardized treatment of measurement uncertainty in metrology is the internationally accepted *Guide to the Expression of Uncertainty in Measurement* (GUM) [22,23] which allows both, random and systematic errors, to be treated consistently. However, the GUM is not directly applicable to the analysis of dynamic measurements. Therefore, several approaches have been made in recent years to extent uncertainty evaluation in line with the GUM to the case of dynamic measurements [13–21]. While these approaches mainly resort to techniques from DSP, they also differ from them to some extent accounting for the particular requirements of uncertainty evaluation guide lines in metrology [18,21]. One of the differences is that according to supplement 1 to the GUM [23] the uncertainty is obtained as the standard deviation of a (degree-of-belief) probability density function (PDF) for the measurand, rather than as an estimate of a standard deviation of a sampling distribution. This point of view enables to consistently include also the treatment of systematic influences which, in metrology, are often most important.

For the particular model (1) recently two approaches have been proposed for the compensation of dynamic effects in terms of an IIR [1] and an FIR [14] compensation filter. The FIR approach uses numerical means to design a digital filter with compensation in the passband and attenuation in the stop band. The IIR approach simply inverts model (1) and accompanies this by an appropriate analogue IIR-type low-pass filter (here discretized for discrete-time processing). For both types of digital filters real-time capable schemes for the evaluation of uncertainty in line with the GUM have been proposed recently [15,17,19]. The uncertainty evaluation approach for the IIR compensation filter is based on linearization and employs a state-space representation while the approach for the FIR filter does not require linearization and can be implemented in terms of a digital filter.

The goal of this paper is to compare the performance of the two particular approaches [1,14] for dynamic error compensation in terms of the resulting uncertainty. The comparison is made by using simulations which allow for the assessment of the various uncertainty sources. The construction and application of an FIR compensation filter typically requires more effort compared to the considered IIR filter approach. On the other hand, for IIR filters [1] the phase response of the compensated system usually is nonlinear [24] which may result in compensation errors. Our main conclusion is that both approaches may well be applied but that the uncertainty of the IIR filter approach is larger due to compensation errors.

## 2. Compensation Task and Considered Digital Compensation Filters

**Figure 1.**
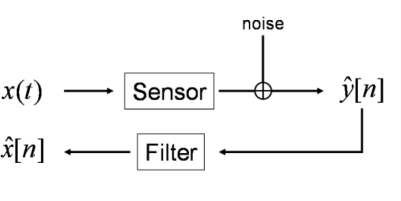
Measurement task of sensor compensation by digital filtering.

We consider the following measurement task: a continuous-time input signal *x*(*t*) (the time-dependent physical quantity to be measured) acts as input to a sensor with system model (1). The corresponding continuous-time output signal *y*(*t*) is discretized by an analogue-to-digital converter. We model discretization (and possible further) errors as additive stationary white noise *ɛ*[*n*] with known variance, resulting in the available data *ŷ*[*n*] = *y*(*nT_S_*) + *ɛ*[*n*], where *f_S_* = 1/*T_S_* denotes the chosen sampling frequency. Estimates *x̂*[*n*] of the discrete-time input signal *x*[*n*] are calculated by applying a digital deconvolution filter, see Figure 1.

We consider the two recently proposed approaches [1] and [14] for the construction of the deconvolution filter. The first directly inverts the continuous model (1) and results in an analogue IIR filter (here subsequently discretized) while the second employs a linear least squares fit in the frequency domain yielding a digital FIR filter from the start. Note that the considered FIR approach requires an additional time sample delay.

## 3. Uncertainty Evaluation Methods

We describe uncertainty evaluation in line with the GUM and briefly recall the two considered uncertainty evaluation methods for FIR and IIR filtering.

We assume that the characterization of the sensor in terms of calibration measurements provides parameter estimates *δ̂*, *ω̂*_0_, *Ŝ*_0_ for the system (1) with an uncertainty matrix *U*(*δ̂*, *ω̂*_0_, *Ŝ*_0_), see [14]. This uncertainty matrix can be interpreted as the covariance matrix of a joint Gaussian PDF, *cf*. [23]. In order to calculate the uncertainty caused by the uncertainty of the system, this uncertainty has to be propagated through the filter design. This results in the uncertainty matrix ***U_θ̂_*** of the filter coefficient vector, where **θ** stands for the filter coefficients of the deconvolution filter, see [23]. Once the uncertainty matrix ***U_θ̂_*** has been derived its contribution to the uncertainty of the corresponding estimate *x̂*[*n*] of the input signal can be utilized as described below.

In addition to ***U_θ̂_***, signal noise and non-perfect compensation influence the resulting uncertainty associated with *x̂*[*n*]. The contribution of signal noise is calculated by propagating the covariance of the noise through the compensation filter, see [15,17]. The non-perfect compensation due to regularization or non-perfect construction of the deconvolution filter results in remaining dynamic errors:

(2) $$\Delta [n]=y_{\text{comp}}[n+n_{0}]-x[n]$$

between the output of the compensation filter *y*_comp_[*n*] = (*g* * *y*)[*n*] and the actual, unknown input of the sensor; *n*_0_ denotes a possible known time sample delay. Utilizing the well-known inequality for the Fourier transform *F* (Ω) of a function *f* (*t*):

(3) $$\left|f\left(t\right)\right|\leq \int_{-\infty }^{\infty }\left|F\left(\Omega \right)\right|d\Omega$$

we can derive an upper bound on the dynamic error Δ[*n*] by assuming knowledge about an upper bound |*X̄*(Ω)| on the continuous-time input signal magnitude spectrum |*X*(*j*Ω) |≥| *X̄*(Ω)|, where Ω = *ωf_S_* with *f_S_* denoting the chosen sampling frequency. The resulting bound is given by:

(4) $$\left|\Delta \left[n\right]\right|\leq \frac{1}{2\pi }\int_{-\pi f_{S}}^{\pi f_{S}}\sum_{k}|\bar{X}\left(\Omega +2\pi kf_{S}\right)|\cdot \left|e^{j\Omega /f_{S}n_{0}}G\left(e^{j\Omega /f_{S}}\right)H\left(j\left(\Omega +2\pi kf_{S}\right)\right)-1\right|d\Omega =:\bar{\Delta }$$

where *G*(*e^jΩ/f_S_^*) denotes the frequency response of the compensation filter (realized by either an FIR or IIR filter), see [18,19]. Note that the upper bound Δ̄ is time-independent, and it is similar to a corresponding continuous-time result given in [13].

In order to determine the contribution of the dynamic errors to the uncertainty *u*(*x̂*[*n*]), a PDF is assigned which encodes the available knowledge about the dynamic errors. According to the supplement 1 to the GUM [23] a uniform PDF within the interval [Δ̄,Δ̄] results in our case, where Δ̄ denotes the upper bound (4). The resulting standard uncertainty, obtained as the standard deviation of this PDF, is given by:

(5) $$u(\Delta )=\frac{\bar{\Delta }}{\sqrt{3}}$$

The overall dynamic uncertainty is then evaluated according to:

(6) $$u^{2}(\hat{x}[n-n_{0}])=\text{var}\left(\left(g*y)[n\right]\right)+\frac{{\left(\bar{\Delta }\right)}^{2}}{3}$$

where the variance on the right-hand side takes into account the uncertainty of the filter coefficients of *g*(*z*) and the variance of the noise.

### 3.1. Uncertainty evaluation for IIR filtering

For the evaluation of the uncertainty *u*(*x̂*[*n*]) associated with *x̂*[*n*] calculated by IIR filtering of the noisy sensor output signal *ŷ*[*n*] according to:

(7) $$\hat{x}[n]=\sum_{k=0}^{p}b_{k}\hat{y}[n-k]\;-\;\sum_{k=1}^{p}a_{k}\hat{x}[n-k]$$

an explicit expression for the variance on the right-hand side of (6) has been derived in [17] utilizing a state-space form. The resulting uncertainty in (6) is then given by:

(8) u2(x^[n])=ΦT(n)Uθ^Φ(n)+∑r,sg[r]g[s]u(y^[r],y^[s])+Δ¯23

where *g*[*r*] denotes the impulse response of the compensation filter *g*(*z*) and the expression:

(9) $$\Phi (n)={\left(\begin{array}{ccc} \frac{\partial \hat{x}[n]}{\partial \theta_{1}} & \cdots & \frac{\partial \hat{x}[n]}{\partial \theta_{2p+1}} \end{array}\right)}^{T}$$

denotes the vector of first order derivatives of the estimate with respect to the elements of the filter coefficient vector. The calculation scheme (8) is real-time capable as for (9) a corresponding update relation is available, *cf*. [17].

### 3.2. Uncertainty evaluation for FIR filtering

For an uncertainty evaluation in the context of FIR filtering the variance term in (6) can be calculated in a straightforward way, see [14,15], leading to:

(10) u2(x^[n])=θ^TUylowθ^+y^low[n]TUθ^y^low[n]+Tr(UylowUθ^)+Δ¯23

where *Tr* denotes the trace of a square matrix and **ŷ**_low_[*n*] = (*ŷ*_low_[*n*],...., *ŷ*_low_[*n* − *N*_comp_])^T^; *ŷ*_low_ denotes the low-pass filtered sensor output signal and **U**_y_low__ stands for the covariance matrix of ***ŷ***_low_[*n*]. For stationary noise only the second term on the right-hand side of (10) is time-dependent and the uncertainty evaluation can be realized at low computational costs during the measurement.

## 4. Results

We compare the two compensation filter methods [1] and [14] in terms of the resulting uncertainties obtained by applying the above described uncertainty evaluation schemes for FIR and IIR filtering. To this end, simulations are employed using the following values of system parameters for model (1):

(11) $$\theta ={\left(\delta ,f_{0},S_{0}\right)}^{T}:={\left(8.3\cdot 10^{-3},\;29.4\cdot 10^{4}\;\text{kHz},\;0.985\right)}^{T}$$

which are related to parameters of a typical accelerometer. For the construction of the compensation filters uncertain knowledge about the system (1) was modeled by assuming that the following parameter estimates including their uncertainty matrix were available:

(12a) θ^=(δ^,f^0,S^0)T:=(0.01,3⋅104 kHz,1)T

(12b) Uθ^=diag(0.1 δ^,0.03 f^0, 0.01 S^0)

**Figure 2.**
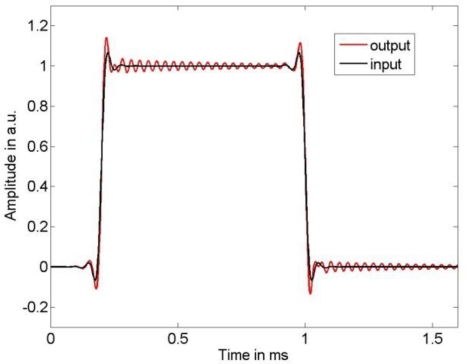
Narrow-banded sensor input signal and resulting sensor output signal.

**Figure 3.**
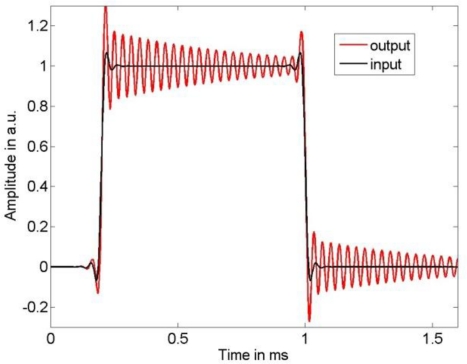
Broad-banded sensor input signal and resulting sensor output signal.

As input signal we chose a low-pass filtered rectangular function, where we employed low-pass filter cut-off frequencies of 10 kHz and 25 kHz to limit the bandwidth of the sensor input signal. The sensor output signal was calculated by a convolution of the chosen input signal with the LTI system transfer function (1) using the parameters in (11). Figures 2 and 3 show the input signal and the resulting sensor output signal. It can be seen that the larger input signal bandwidth results in significant dynamic errors due to the sensor’s resonance frequency. The output signal was thereafter disturbed by additive stationary noise with variances σ^2^ = 1 e−3, σ^2^ = 3 e−4, and σ^2^ = 1 e−6, respectively. As sampling frequency we chose 500 kHz. According to Figure 1, the measurand of this dynamic measurement was the band-limited sensor input signal.

The IIR deconvolution filter was derived according to [1] as a cascade of the inverse of model (1) with parameter vector (12a), and the second-order system:

(13) $$G_{T}(s)=\frac{\omega_{T}^{2}}{s^{2}+2\delta_{T}\omega_{T}s+\omega_{T}^{2}},$$

where we chose the parameters for (13) as $\delta_{T}=1/\sqrt{2}$, *ω_T_* = 120 · *π*kHz.

We discretized this system employing the bilinear transform with frequency pre-warping to meet the resonance frequency, see [24]. The resulting digital filter was employed in cascade with a digital order 4 Butterworth low-pass filter in order to increase noise attenuation. The low-pass cut-off frequency of this filter was set to 30 kHz and 53 kHz for the input signal with bandwidth of 10 kHz and 25 kHz, respectively. The resulting compensation filter and the frequency response of the compensated system are given in Figure 6.

The FIR deconvolution filter was designed according to [14] by means of a least squares fit to the reciprocal frequency response of model (1) with parameter vector (12a) in the frequency region from DC up to 60 kHz. As appropriate filter order we determined 12 with an according time sample delay of 6 samples. For the additional low-pass filter employed in this technique we chose an order 60 FIR filter, designed using the window technique with a Hamming window. The low-pass filter cut-off was taken as 30 kHz and 50 kHz for the input signal with bandwidth of 10 kHz and 25 kHz, respectively. The frequency response of the compensation filter and that of the compensated system are shown in Figure 7.

A comparison of the frequency response of the compensated systems shows that both, FIR as well as IIR filter, yield a good approximation to the inverse of model (1) in the relevant frequency region for the available knowledge about the actual model parameters. While the phase response of the compensated system for the IIR filter is only approximately linear, the FIR filter results in a compensated system with an almost perfect linear phase response that can be realized in the time domain by a sample shift. Thus, the corresponding error bound (4) for the IIR compensation filter is larger than that for the FIR filter. This can be seen in Figures 8 and 9 where the uncertainties associated with the estimation of the narrow-banded and broad-banded input signal are given. In all cases the resulting uncertainties for the IIR compensation filter are larger than those for the FIR compensation filter. The maximum difference between the obtained corresponding uncertainties is about 30%.

**Figure 4.**
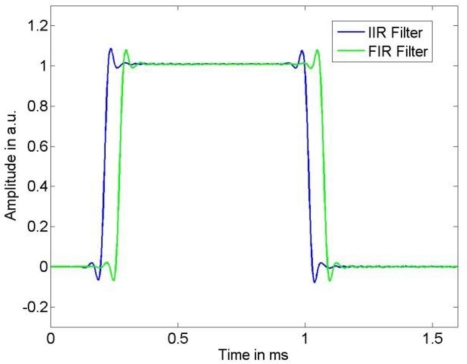
The compensated output signals resulting from the IIR and the FIR compensation filter for the narrow-banded sensor input signal.

**Figure 5.**
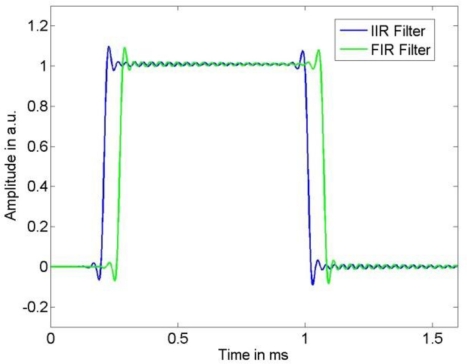
The compensated output signals resulting from the IIR and the FIR compensation filter for thebroad-banded sensor input signal.

It appears that the shape of the uncertainties for the FIR and IIR compensation are similar. As expected, for both filter types a larger noise variance results in an increased uncertainty of the input signal estimate. The influence of the model uncertainty, namely the impact of the resonance frequency uncertainty *u*(*f*_0_) and damping uncertainty *u*(*δ*), can be seen especially in Figure 9 as the employed input signal has significant spectrum near the system’s resonance and thus increases. Moreover, it can be seen in Figure 9 that due to the larger cut-off frequencies of the low-pass filters the output signal noise is less attenuated than for the narrow-banded input signal shown in Figure 8. Although these characteristics of the uncertainty are similar for FIR and IIR compensation, the larger value of the error bound (4) for the IIR compensation filter causes the larger uncertainty for this filter. On the other hand, as can be seen in Figures 4 and 5, the time delay of the FIR filter result is significantly larger than that of the IIR compensation filter and hence, when speed is an issue, the IIR filter is preferable.

**Figure 6.**
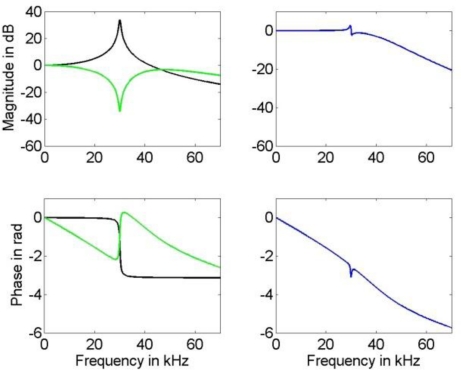
Left: Frequency response of the sensor model (black) with system parameter vector (11) and the IIR compensation filter (green) designed for the available estimate (12a) of the system parameter vector for estimation of the broad-banded (25 kHz) input signal. Right: Frequency response of the actual compensated system.

**Figure 7.**
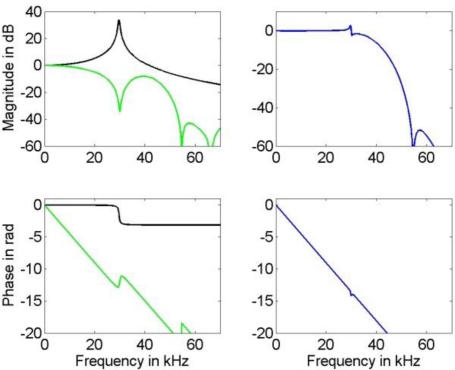
Left: Frequency response of the sensor model (black) with system parameter vector (11) and the FIR compensation filter (green) designed for the available estimate (12a) of the system parameter vector for estimation of the broad-banded (25 kHz) input signal. Right: Frequency response of the actual compensated system.

**Figure 8.**
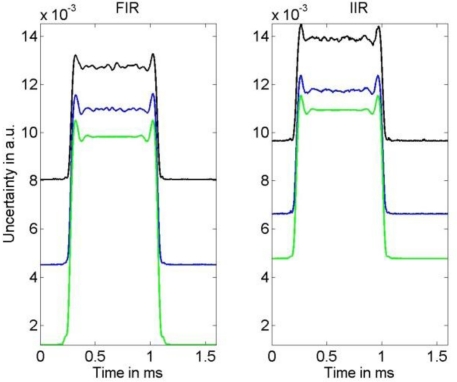
Left: Uncertainty associated with the FIR compensation filter result for three different noise values obtained for the narrow-banded input. Right: Uncertainty associated with the IIR compensation filter result.

**Figure 9.**
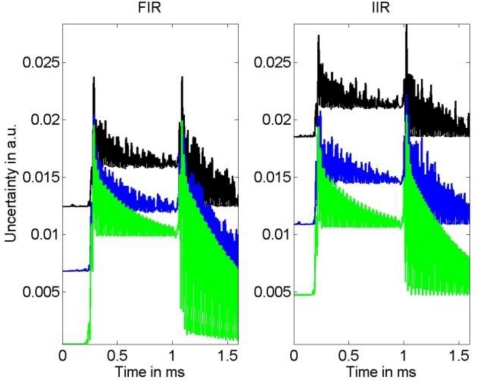
Left: Uncertainty associated with the FIR compensation filter result for three different noise values obtained for the broad-banded input. Right: Uncertainty associated with the IIR compensation filter result.

It should be noted that the frequency responses of the compensated system shown in Figures 6 and 7 are available only for a simulation, as their calculation requires knowledge about the true parameters (11) of the underlying system (1). In an application, the compensated system could be evaluated only approximately by inserting the available parameter estimates (and not their unknown true values) for the system (1). In that case, the approximation of the inverse system would appear ideal also around the resonance frequency, and for the FIR filter the phase of the compensated system as perfectly linear.

## 5. Conclusions

An FIR and an IIR filter approach for the compensation of a second-order system have been compared in terms of resulting uncertainties. The main drawback of the considered IIR filtering approach is the nonlinear phase response of the compensated system which may result in significant enlarged uncertainties. The non-linearity could be eliminated by a bi-directional application of the filter, but this technique is not possible for real-time measurements. We conclude that the considered FIR compensation filter should be preferred as long as the time sample delay introduced for its construction is not critical and real-time evaluation of uncertainties is desired.
